# ACTN4 Mediates SEPT14 Mutation-Induced Sperm Head Defects

**DOI:** 10.3390/biomedicines8110518

**Published:** 2020-11-19

**Authors:** Yu-Hua Lin, Chia-Yen Huang, Chih-Chun Ke, Ya-Yun Wang, Tsung-Hsuan Lai, Hsuan-Che Liu, Wei-Chi Ku, Chying-Chyuan Chan, Ying-Hung Lin

**Affiliations:** 1 Department of Chemistry, Fu Jen Catholic University, New Taipei City 242, Taiwan; jorgesuperowl@gmail.com; 2 Division of Urology, Department of Surgery, Cardinal Tien Hospital, New Taipei City 231, Taiwan; 3 Department of Biological Science and Technology, National Chiao Tung University, Hsinchu 300, Taiwan; bagiao2003@gmail.com; 4 Gynecologic Cancer Center, Department of Obstetrics and Gynecology, Cathay General Hospital, Taipei 106, Taiwan; 5 School of Medicine, Fu Jen Catholic University, New Taipei City 242, Taiwan; Joseph@cgh.org.tw (T.-H.L.); 089052@mail.fju.edu.tw (W.-C.K.); 6 PhD Program in Nutrition & Food Science, Fu Jen Catholic University, New Taipei City 242, Taiwan; koacurtis@gmail.com; 7 Department of Urology, En Chu Kong Hospital, New Taipei City 237, Taiwan; 8 Graduate Institute of Biomedical and Pharmaceutical Science, Fu Jen Catholic University, New Taipei City 242, Taiwan; vic0009@gmail.com (Y.-Y.W.); bo7926@hotmail.com (H.-C.L.); 9 Department of Obstetrics and Gynecology, Cathay General Hospital, Taipei 106, Taiwan; 10 Department of Obstetrics and Gynecology, Taipei City Hospital, Renai Branch, Taipei 106, Taiwan; drobsgyn@seed.net.tw

**Keywords:** male infertility, teratozoospermia, septin, SEPT14, ACTN4

## Abstract

Septins (SEPTs) are highly conserved GTP-binding proteins and the fourth component of the cytoskeleton. Polymerized SEPTs participate in the modulation of various cellular processes, such as cytokinesis, cell polarity, and membrane dynamics, through their interactions with microtubules, actin, and other cellular components. The main objective of this study was to dissect the molecular pathological mechanism of *SEPT14* mutation-induced sperm head defects. To identify SEPT14 interactors, co-immunoprecipitation (co-IP) and nano-liquid chromatography-mass spectrometry/mass spectrometry were applied. Immunostaining showed that SEPT14 was significantly localized to the manchette structure. The SEPT14 interactors were identified and classified as (1) SEPT-, (2) microtubule-, (3) actin-, and (4) sperm structure-related proteins. One interactor, ACTN4, an actin-holding protein, was selected for further study. Co-IP experiments showed that SEPT14 interacts with ACTN4 in a male germ cell line. SEPT14 also co-localized with ACTN4 in the perinuclear and manchette regions of the sperm head in early elongating spermatids. In the cell model, mutated SEPT14 disturbed the localization pattern of ACTN4. In a clinical aspect, sperm with mutant SEPT14, SEPT14^A123T^ (p.Ala123Thr), and SEPT14^I333T^ (p.Ile333Thr), have mislocalized and fragmented ACTN4 signals. Sperm head defects in donors with SEPT14 mutations are caused by disruption of the functions of ACTN4 and actin during sperm head formation.

## 1. Introduction

### 1.1. Male Infertility and Mutations

Male sterility occurs in up to 7% of men of reproductive age worldwide, and in as many as half of these cases, the cause cannot be determined [1,2]. The known major causes of male infertility include anatomic abnormalities, endocrine defects, immunologic dysfunction, infection, Y chromosome deletion, environmental exposure, and gene mutations [3,4,5]. Semen analysis is a critical tool for identifying the cause of infertility, and semen can be classified as normozoospermia, oligozoospermia, asthenozoospermia, teratozoospermia, or azoospermia [6]. Teratozoospermia is frequently accompanied by sperm DNA defects and can have negative effects on pregnancy outcomes and embryo progress, including recurrent spontaneous abortion, pregnancy failure, and lower live birth rates [7,8,9,10,11,12,13]. Several mutations in certain genes have been linked to teratozoospermia, including *PROTAMINE*, *SPATA16*, *AURKC*, *PICK1*, *SEPT12*, *DPY19L2*, and *SEPT14* [14,15,16,17].

### 1.2. Septins and Male Reproduction

Septins (SEPTs) are highly conserved GTP-binding proteins that are the fourth component of the cytoskeleton [18,19]. SEPT proteins polymerize into hetero- and homo-oligomeric structures and modulate various cellular processes, including cell polarity, cytokinesis, and membrane dynamics, via their interaction with microtubules, actin, and phospholipid membranes [18,19,20,21,22]. Dysregulation of human SEPTs has been linked to the molecular pathology of several diseases, including leukemia, neurological illnesses, and male sterility [23,24]. SEPT4 and SEPT7 are biomarkers for human asthenozoospermia and teratozoospermia, respectively [25,26,27]. In addition, *Sept4*-null male mice are infertile, with immotile sperm and defective sperm tails [28,29]. Our previous studies revealed that *SEPT12* is a testis- and post-meiotic-specific gene, and sperm from *Sept12*-defective mice showed sperm head and tail defects [30]. Furthermore, the development of murine embryos fertilized with *Sept12-*deficient sperm through ICSI was arrested at the early morula stage [31]. Mutated SEPT12 also causes impaired sperm heads, bent tails, and DNA damage [16,30,32].

### 1.3. SEPT14

SEPT14 was originally identified as a testis-enriched protein that interacts with SEPT9 [33]. SEPT14 is required for cortical neuronal migration through its interaction with SEPT4 [34]. In addition, two genetic variants of *SEPT14* have been identified as associated with a reduced risk of Parkinson’s disease [35]. In the male reproductive system, SEPT14 is mainly localized at the sperm head and tail [36]. SEPT14 expression in testicular tissues is lower in infertile men with hypospermatogenesis, maturation arrest, and Sertoli cell-only syndrome than in fertile men [37]. Recently, we identified two mutations of *SEPT14* in teratozoospermia donors, SEPT14^A123T^ (p.Ala123Thr) and SEPT14^I333T^ (p.Ile333Thr). Sperm from the mutated donors showed a high percentage of defects in the sperm-head (90 ± 4%) and high levels of sperm nuclear damage [38]. In addition, the mutant SEPT14 proteins disturbed the polymerization ability and co-localization of F-actin filaments in vitro [38]. In this study, the main objective was to dissect the molecular mechanism underlying the pathological effects of mutated SEPT14 leading to sperm-head defects.

## 2. Materials and Methods

### 2.1. Separation and Isolation of Testicular Germ Cell Populations

The animal study was approved by the Institutional Animal Care and Use Committee of Fu-Jen Catholic University (A10577, approved date: 17 March 2017). Testes were isolated from adult mice (C57BL/6, *n* = 3; postnatal day > 80). The isolation and separation protocols used were similar to those used in previous studies [39,40]. After de-capsulation of the testes, the seminiferous tubules were handled in DMEM/F12 medium with a mixture of digestion enzymes. The samples were incubated for 1.5 h at 37 °C with rotation at 140 rpm. The samples were filtered through 35 μM nylon filters (Falcon; Becton Dickinson) and centrifuged. After centrifugation at 700× *g*, 400× *g*, 200× *g*, and 100× *g*, four suspension solutions were collected. Finally, the cells were collected from these suspensions by centrifugation at 3000× *g*. The pellets were suspended in 1× PBS and spread on a slide. Mature spermatozoa were collected from the cauda epididymis of adult mice. After air-drying, the slides were stored at −80 °C for immunofluorescence assays.

### 2.2. Human Sperm Collection and Immunofluorescence Assay

This study was approved by the Ethics Committee of Cathay General Hospital (IRB Approval No.: CGH-P102031). All the collected protocols have been described in our previous study. All recruited participants signed an informed consent form. Semen samples were obtained by masturbation after 3–5 days of sexual abstinence. After liquefying the semen at room temperature, routine semen analysis was performed according to the WHO 2010 criteria. After washing with 1XPBS, the sperm were spread on the slides and air-dried. The slides were treated with 0.1% Triton X-100, washed twice with Tris-buffered saline (TBS), and then incubated with SEPT14 (Proteintech, Cat No, 24590-1-AP) and α-tubulin (GeneTex, Cat No. GTX628802), and ACTN4 (GeneTex, Cat. No. GTX15648; Abcam, Cat. ab108198) antibodies for 60 min at 25 °C. After washing with TBS, the sections were incubated with secondary antibodies for 60 min at room temperature and then washed again with TBS. 4′,6-Diamidino-2-phenylindole (DAPI) was used to stain the nuclei. The Leica DM 2000 microscope was used for observation, and the images were acquired using the SPOT 5.0 software. All steps were performed according to our previous studies [41,42].

### 2.3. Cloning and Transfection

To generate human testicular complementary DNA (cDNA), human testicular RNA was obtained from a human RNA panel (Clontech, Mountain View, CA, USA). Total testicular cDNA was generated from this RNA by Superscript^™^ III Reverse Transcriptase (Invitrogen), which was stored at −20 °C until use, as described in our previous study [39]. Full-length *SEPT14* transcripts were amplified and cloned into the pFLAG-CMV2 plasmid. The construct was confirmed by Sanger sequencing. Next, NTERA-2 cl.D1 (NT2D1) cells (ATCC, Manassas, VA, USA), a pluripotent human testicular embryonal carcinoma cell line, or HeLa cells were transfected with these plasmids using Lipofectamine reagent (Cat No.: 11668; Invitrogen, Carlsbad, CA, USA). The protocols used were published in our previous studies [38,43]. Total cell lysates were then collected for co-immunoprecipitation (co-IP).

### 2.4. Co-Immunoprecipitation

Co-IP was performed according to the manufacturer’s protocol and the methods used in our previous study [43,44,45]. Total cell lysate containing 4 mg of protein in 1 mL of lysis buffer was precleared by incubation with 50 µL of protein A/G beads (Santa Cruz Biotechnology, Santa Cruz, CA, USA) for 1 h at 4 °C on a rotator (15 rpm). Next, the precleared supernatants were collected by centrifugation at 1000*× g* for 30 s at 4 °C. The supernatants were incubated with either the control IgG or a FLAG antibody (Cat No.: A5441; Sigma) at 4 °C on a rotator (15 rpm) overnight. Next, the samples were centrifuged at 1000× *g* for 30 s at 4 °C. The immunoprecipitated samples were collected and washed twice with 1× phosphate-buffered saline (PBS). Then, the immunoprecipitated samples were immunoblotted (IB) using a primary FLAG antibody (Cat No.: 8127; Cell Signaling Technology, Boston, MA, USA). After confirming the IB results, the immunoprecipitated samples were transferred to the proteomic core (Tzong Jwo Jang Mass Spectrometry Laboratory, School of Medicine, Fu Jen Catholic University) for MS analysis.

### 2.5. Mass Spectrometry Analysis

The immunoprecipitated mixtures were reduced with dithiothreitol, *S*-alkylated with iodoacetamide, and treated with Lys-C and trypsin as described in our previous study [46]. The digested peptides were desalted with SDB-XC StageTip (3M Company, MN, USA), followed by SCX StageTip (3M Company) [47]. Then, the products were examined by liquid chromatography-mass spectrometry/mass spectrometry (LC-MS/MS) using a Dionex Ultimate 3000 RSLC nanosystem (Thermo Fisher Scientific, Waltham, MA, USA) and an LTQ Orbitrap XL mass spectrometer (Thermo Fisher Scientific). Finally, protein identification was performed as described in our previous studies [44,48].

## 3. Results

### 3.1. Dynamic Expression of SEPT14 during Murine Sperm Head Formation

**Figure 1:**
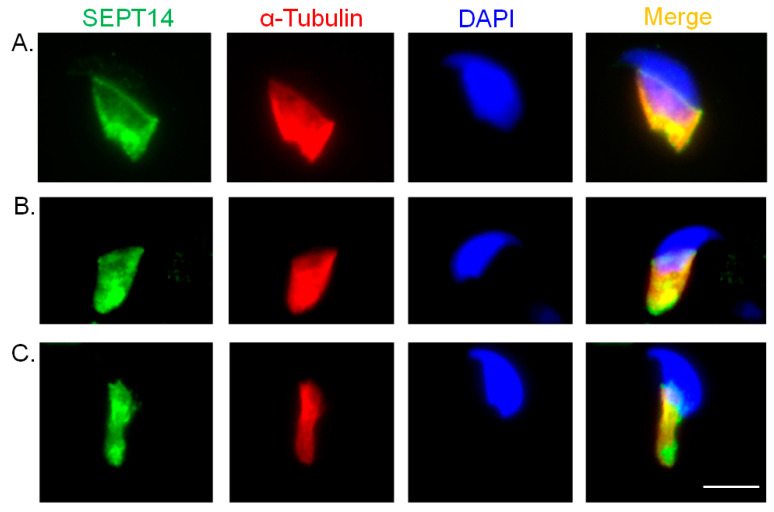
Immunofluorescence detection of SEPT14 during murine sperm head shaping. SEPT14 signals showed multiple co-localizations with the manchette marker, α-Tubulin, in spermatids at step 8 (**A**), 9 (**B**), and 10 (**C**). SEPT14 (green), α-Tubulin (red), and DAPI (blue) stained images are shown, as well as merged images of SEPT14, α-Tubulin, and DAPI signals in spermatids. More than 10 male germ cells have been detected at different stages. Magnification, 1000×. Scale Bar: 10 μm.

Our previous study revealed that sperm from patients with SEPT14^A123T^ and SEPT14^I333T^ mutations exhibited severely malformed heads and DNA fragmentation [38]. However, the expression patterns of SEPT14 during sperm head formation remain unknown. To determine the detailed and dynamic localization of SEPT14 during sperm head formation, murine spermatids were obtained from testicular tissue, and sperm heads from different stages of development were isolated and analyzed by IFA. SEPT14 was initially mainly localized to the perinuclear rim, and manchette structure, a transition structure for sperm head shaping that consists of tubulin and actin in early elongating spermatids during murine spermiogenesis (Figure 1A). Over the course of sperm head shaping, SEPT14 gradually became concentrated in the narrow manchette (Figure 1B,C). These findings suggest that SEPT14 is involved in sperm head shaping during spermiogenesis.

### 3.2. Identification of SEPT14 Interactors in Male Germ Cells

**Figure 2:**
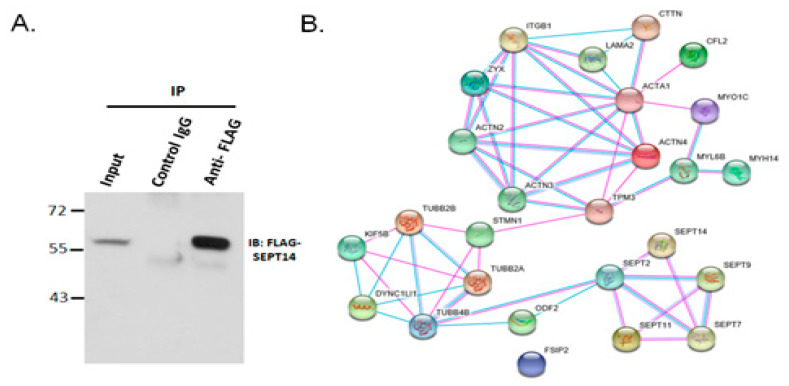
Identification of SEPT14 interactors through co-IP and nano-LC-MS/MS. (**A**) Co-IP analysis of lysates from cells transfected with the pFLAG-SEPT14 plasmid. Lysates were immunoprecipitated with either a nonspecific immunoglobulin G (IgG) control (control IgG) or FLAG antibody (Anti-FLAG). Input protein (5%) was used as an IB control (input). IB revealed a FLAG-SEPT14 signal. The transfected cells have been performed Co-IP and nano- LCMS/MS, triplicate. (**B**) Clustering of SEPT14 interactors. The interaction network of SEPT14 interactors includes (1) SEPT-, (2) actin-, (3) microtubules-, and (4) sperm structure-related proteins, and was generated using STRING software. Green and pink lines indicate interactions elucidated from the previous curated database and the current experimental analysis, respectively.

To dissect the possible molecular mechanism underlying mutated *SEPT14*-caused sperm head defects, SEPT14 interactors were identified by co-*IP* and nano-LC-MS/MS. After the male germ cell line (NT2D1) was transfected with the pFLAG-SEPT14 plasmid, cell lysates were co-immunoprecipitated with the FLAG antibody or mouse immunoglobulin G (IgG). The specific binding was determined by IB using the FLAG antibody (Figure 2A). Next, the interactors were subjected to nano-LC-MS/MS analysis. The interacting proteins were filtered and grouped as follows: (1) SEPT-, (2) microtubule-, (3) actin-, and (4) sperm structure-related proteins, based on the possible cytoskeletal and spermatogenic functions of SEPT14 (Table 1). The interaction networks of these proteins were subsequently generated using STRING (Figure 2B). The interaction networks of SEPT14 revealed a dense and connective relationship when constructed using data from either the previous database (green lines) or experimental analysis (pink lines). Based on these results, we concluded that the SEPT14-interactors were successfully identified; these results show a solid and mutual interaction elucidated using co-IP, LC-MS/MS, and bioinformatics assays.

### 3.3. SEPT14 Interacts and Co-Localizes with ACTN4

**Figure 3:**
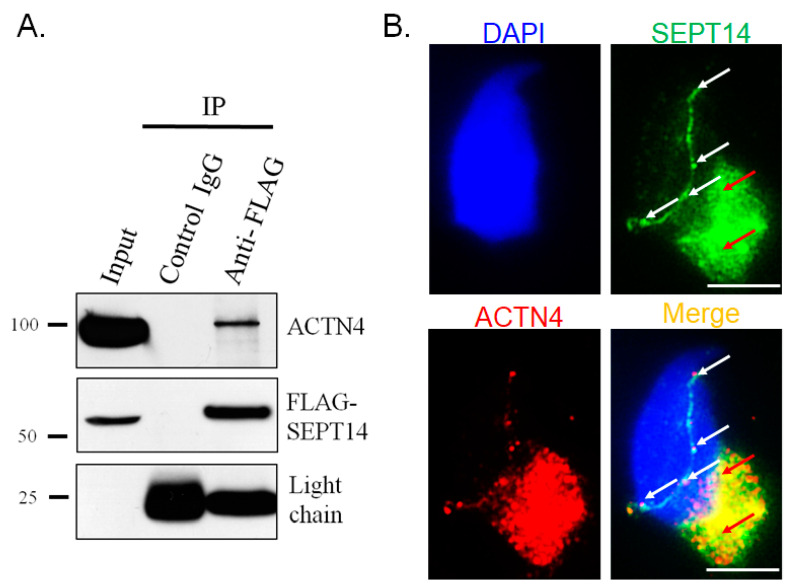
SEPT14 interacts with ACTN4 in male germ cells. (**A**) Co-IP of FLAG-SEPT14 with ACTN4. Lysates from cells transfected with a pFLAG-SEPT14 vector were immunoprecipitated with either a nonspecific control IgG (Lane 2) or FLAG antibody (Lane 3), followed by immunoblotting with an ACTN4 or FLAG antibody. Light chain was used as a loading control. Input protein (5%) was used as an immunoblotting control (Input). (**B**) SEPT14 was co-localized with ACTN4 in murine spermatids. DAPI staining (blue), SEPT14 (green), ACTN4 (red), and a merged image of the DAPI, SEP14, and ACTN4 signals in murine spermatids are shown. SEPT14 signals are near the perinucleus rim (white arrows) and manchette structure (red arrows). More than 10 elongating spermatids have been stained. Magnification, 1000×. Scale Bar: 10 μM.

In our previous study, we found that overexpressed SEPT14 co-localized with polymerized actin in cells [38]. We also identified actin-regulator proteins, which were SEPT14-interactors (Table 1). We looked at three actin holding proteins (ACTN2, ACTN3, and ACTN4) and found that one, ACTN4, was localized to the sperm head, with only a slight presence along the flagella [49,50]. Furthermore, *ACTN4* transcripts have been found to be related to the DNA integrity of boar sperm through RNA sequencing analysis [51]. To evaluate whether SEPT14 interacts with ACTN4, a co-IP assay was performed in a male germ cell line (NT2D1). The cells were transfected with the pFLAG-SEPT14 plasmid and subjected to IP with either an IgG control (Figure 3A, Lane 2) or a FLAG antibody (Figure 3A, Lane 3). Next, the IP samples were immunoblotted with FLAG (Figure 3A, FLAG-SEPT14) and ACTN4 (Figure 3A, ACTN4) antibodies. As shown in Figure 3A, FLAG-SEPT14 was pulled down with the FLAG antibody (Figure 3A, Lane 3, FLAG-SEPT14). The IP sample was also immunoblotted with an ACTN4 antibody (Figure 3A, Lane 3, ACTN4). To confirm whether SEPT14 co-localized with ACTN4 during sperm head formation, IFA was performed. Figure 3B shows that SEPT14 was mainly co-localized with ACTN4 between the perinuclear and manchette regions (Figure 3B, white arrows), as well as in the manchette structure (Figure 3B, red arrows), in early elongating spermatids during murine spermiogenesis (Figure 3B). These findings suggest that SEPT14 interacts and co-localizes with ACTN4 and is involved in murine sperm head formation.

### 3.4. Mutant SEPT14 Proteins Disturb the ACTN4 Localization Pattern but Do Not Affect the Binding Ability of ACTN4

**Figure 4:**
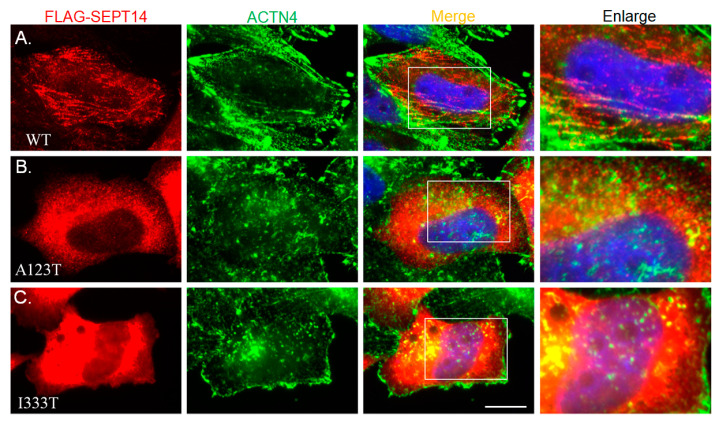
Mutated SEPT14 affects the filament structure of ACTN4. (**A**–**C**) Immunofluorescence staining of cells transfected with plasmids encoding WT FLAG-SEPT14 (WT panel), FLAG-SEPT14^A123T^ (A123T panel), and FLAG-SEPT14^I333T^ (I333T panel) with FLAG and ACTN4 antibodies. From left to right: Images of FLAG antibody (FLAG-*SEPT14*), ACTN4 antibody (ACTN4), and DAPI staining and merged images with FLAG antibody, ACTN4 antibody, and DAPI staining (Merge), as well as an enlarged image (Enlarge). Magnification, 1000×. Scale Bar: 10 μM.

**Figure 5:**
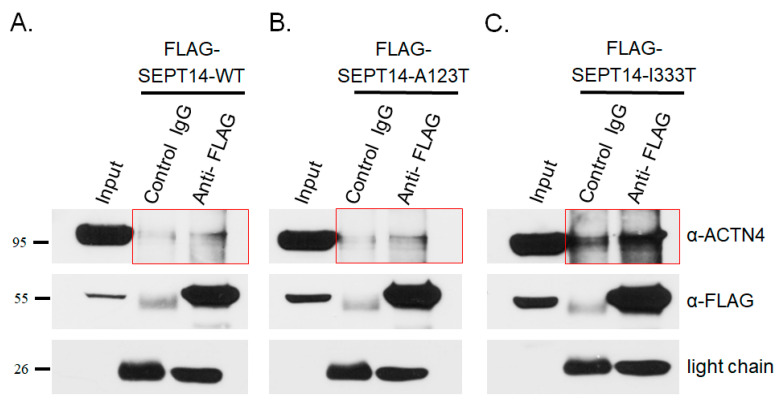
Mutated SEPT14 does not affect the formation of SEPT14/ACTN4 complexes. Co-IP analysis of the binding between SEPT14 and ACTN4. SEPT14-WT, SEPT14-A123T, and SEPT14-I333T are shown in Figures (**A**, **B**, and **C**), respectively. Lysates of HeLa cells transfected with pFLAG-SEPT14 (WT or mutant) were subjected to IP with nonspecific control IgG (Control IgG) or a FLAG antibody (Anti-FLAG) and then immunoblotted with a FLAG (α-FLAG) or ACTN4 (α-ACTN4) antibody. Input protein (5%) was used as a control for immunoblotting of the transfected cell lysates (Input). The light chain was used as a loading control.

In our previous study, we found that the mutant SEPT14 variants, SEPT14^A123T^ (p.Ala123Thr) and SEPT14^I333T^ (p.Ile333Thr) caused teratozoospermia [38]. Here, we showed that SEPT14 interacts with the actin-holding protein ACTN4 (Figure 3). To determine whether the SEPT14^A123T^ and SEPT14^I333T^ mutations interfere with ACTN4 localization, constructs expressing these mutant proteins were transfected into HeLa cells, which lack endogenous SEPT14 expression, to avoid interference by IFA. The cells transfected with wild-type pFLAG-SEPT14 showed filamentous localization patterns (Figure 4A). Co-immunostaining with an ACTN4 antibody showed that the ACTN4 localization pattern was similar to wild-type FLAG-SEPT14 (Figure 4A). In addition, cells transfected with constructs expressing mutated SEPT14^A123T^ and SEPT14^I333T^ showed similar patterns, indicative of a loss of polymerization ability (Figure 4B,C; Left panels). Expression of the mutant SEPT14 disturbed the ACTN4 localization pattern (Figure 4B,C; Right panels). To determine whether the SEPT14^A123T^ and SEPT14^I333T^ mutations affect the binding ability with ACTN4, co-IP analysis was performed. Cells were co-transfected with wild-type and mutant pFLAG-SEPT14 vectors, and cell lysates were subjected to co-IP assay. Figure 5 shows that the binding ability of the mutant SEPT14 variants (SEPT14^A123T^ and SEPT14^I333T^; Figure 5B,C; marked region) with ACTN4 were comparable with that of the wild-type FLAG-SEPT14 (Figure 5A; marked region). These results indicate that these *SEPT14* mutations affect ACTN4 expression patterns, but do not affect the binding ability of ACTN4.

### 3.5. ACTN4 Patterns Are Disturbed in Human Spermatozoa Bard with the Mutated SEPT14

**Figure 6:**
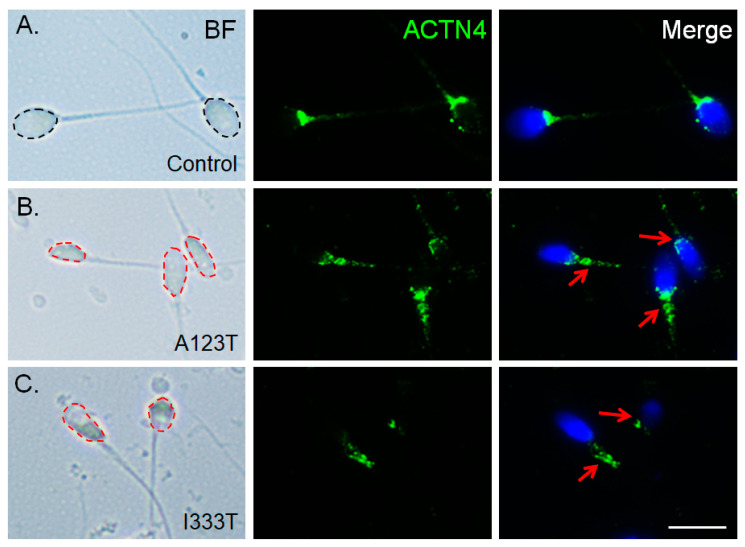
Immunofluorescence staining of ACTN4 in sperm from donors with *SEPT14* mutations. (**A**–**C**) From left to right: bright field (BF), ACTN4 antibody staining (green), and merged DAPI and ACTN4 antibody staining. The figures from top to bottom show spermatozoa from donors with (**A**) WT-SEPT14 (Control), (**B**) SEPT14^A123T^ (A123T), and (**C**) SEPT14^I333T^ (I133T). The black and red dashed lines around the sperm indicate normal and abnormal sperm head morphologies, respectively. The red arrows indicate fragmented ACTN4 signals. Scale Bar: 50 μM.

To determine whether the *SEPT14* mutations affect ACTN4 in vivo, spermatozoa from donors with mutated SEPT14^A123T^ (p.Ala123Thr) and SEPT14^I333T^ (p.Ile333Thr) were immunostained with the ACTN4 antibody. Figure 6 shows that sperm with mutated *SEPT14* had defective sperm heads (Figure 6B,C) compared to wild-type *SEPT14* sperm (Figure 6A). Staining with an ACTN4 antibody showed fragmented and mislocalized ACTN4 signals in sperm with mutant SEPT14 (Figure 6B,C, red arrows). These data demonstrate that mutated SEPT14 also interrupts the localized patterns of ACTN4 in vivo.

## 4. Discussion

In our previous study, through screening of male infertility cases, we showed that *SEPT14* mutations result in sperm head defects and the mutated SEPT14 disturbed the polymerized patterns of F-actin. In this study, we identified SEPT14 interactors through co-IP and nano-LC-MS/MS. ACTN4, an actin regulator, was also identified. During sperm head formation, ACTN4 co-localizes with SEPT14. In addition, mutated SEPT14 disturbed the filamentous localization patterns of ACTN4. In clinical aspects, the human sperm with the mutated SEPT14 also interrupts ACTN4 localization in vivo. Based on these findings, we propose that SEPT14/ACTN4 complexes play a critical role in sperm head formation during human spermiogenesis.

### 4.1. Identification of the SEPT14 Interactor ACTN4 through a Proteomic Assay

This is the first study, to our knowledge, to characterize a possible pathological mechanism of mutated SEPT14-induced sperm head defects by identifying SEPT14 interactors through co-IP and nano-LC-MS/MS. Four categories of interactors, which were based on their molecular functions in sperm head development, were: (1) SEPT-, (2) microtubule-, (3) actin-, and (4) sperm-related proteins [52,53]. As we showed that SEPT14 proteins formed actin-like filament structures in our previous study, we focused on actin-related interactors [38]. There are two groups of actin-related interactors: (1) regulators of actin polymerization (CFL2, TPN3, and ZYX) and (2) ACTN group proteins (ACTN2–4; Table 1). In this study, we identified more than twenty SEPT14-interactors through co-IP and LC-MS/MS; the sensitivity of this method limited the identified number of interactors. The identified SEPT14-interactors include direct interactors, and those which interact indirectly, through the formation of protein complexes. The primary function of ACTN proteins is to hold actin filaments together [50,54], and several studies have indicated that ACTN4 is significantly correlated with sperm morphology. In guinea pig sperm, ACTN4 was found to be localized to the sperm head, with only a slight presence along the flagella [49]. In addition, RNA sequencing has shown that *ACTN4* transcripts are related to DNA integrity in the sperm head of boars [51]. Third, mutations and abnormally elevated expression levels of actinin-4 have been linked to kidney disorders (e.g., focal segmental glomerulosclerosis and minimal-change nephrotic syndrome), and a large number of these cases (6/8, for minimal-change nephrotic syndrome) were accompanied by high levels of teratozoospermia [55,56,57]. Based on these characteristics of ACTN4, we selected it for further study.

### 4.2. SEPTs Interact with ACTIN in Cells

SEPTs interact with actin, microtubules, and phospholipid membranes [18,58]. These interactions affect various cell processes, including cell cytoskeleton modulation, cytokinesis, and cell compartmentalization. Several proteins are involved in regulating the interaction between SEPTs and actin. (1) The filamentous structure of SEPT2/6/7 complexes is associated with actin stress fibers through anillin [20,59]. Moreover, depletion of SEPT2 and 7 by siRNA disturbs actin stress fibers in NIH3T3 cells. (2) In interphase and dividing cells, SEPT2 interacts with myosin II, and loss of this interaction also disturbs actin stress fibers, leading to mitotic arrest in CHO-K1 cells [60]. (3) SEPT2/6/7 regulates actin organization through the SOCS7/NCK pathway in HeLa cells [61]. Knockdown of SEPT2, 6, and 7 in cells disrupts stress fibers and cell polarity. However, whether and how SEPT14 regulates actin is currently unknown. In our previous study, SEPT14 was found to co-localize as a long, thin filament with actin, and mutated SEPT14 disturbed actin stress fibers in HeLa cells [38]. In this study, ACTN4, an actin-holding protein, was shown to be associated with the interaction between SEPT14 and actin (Table 1 and Figure 3). Mutated SEPT14 damaged the stress fiber-like pattern of ACTN4, which was similar to that of actin stress fibers (Figure 4) [38]. Importantly, damaged patterns were also observed in SEPT14-mutated sperm (Figure 6). Based on these results, we propose that SEPT14 may regulate actin stress fibers through ACTN4 in male germ cells.

### 4.3. Molecular Roles of SEPT14/ACTN4 Complexes during Sperm Head Shaping

**Figure 7:**
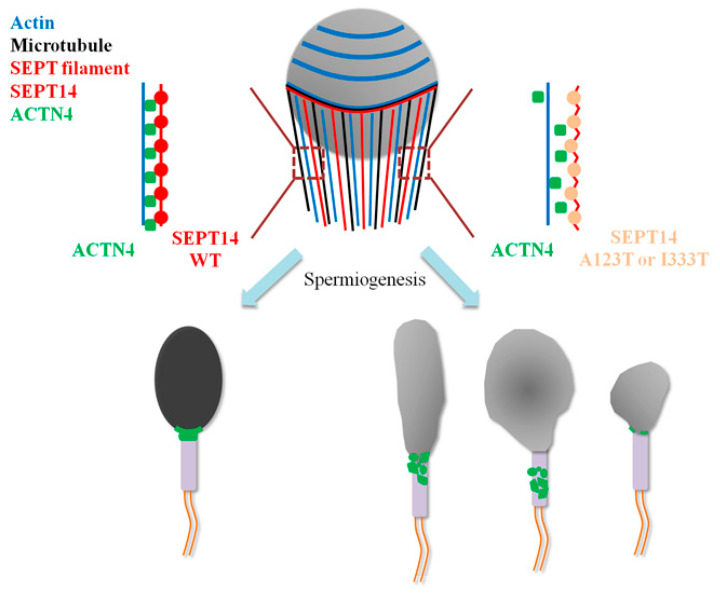
Graphical illustration of the molecular roles of SEPT14, ACTN4, and ACTIN in sperm head formation. During sperm head formation, the manchette structure, which consists primarily of microtubules and actin, assists with sperm head shaping. Filamentous SEPT14 binds to ACTN4/ACTIN complexes, which are involved in sperm head shaping. In sperm with SEPT14 mutations (e.g., A123I or I333T), mutated SEPT14 affects ACTN4-ACTIN function, resulting in abnormal sperm head morphology.

During sperm head shaping, the manchette structure supports the processes of nucleus shaping and cytoplasm removal [52,53]. The manchette is a transient structure organized by cytoplasmic microtubules, and actin that starts developing in step 8 and disappears in step 16 of murine spermiogenesis [62]. By screening the coding region of SEPT14 in donors of male infertility (*n* = 254) and normal controls (*n* = 116), six donors with SEPT14 mutations, SEPT14^A123T^ (*n* = 3), and SEPT14^I333T^ (*n* = 3) were identified, which showed morphological defects of the sperm head [38]. In this study, the dynamic localization of SEPT14 in the manchette structure was observed during murine sperm head formation (Figure 1). During the process of sperm head shaping, SEPT14 and ACTN4 showed the same patterns (Figure 3B). From a clinical perspective, sperm from donors with SEPT14 mutations also showed ACTN4 disruption (Figure 6). Possible working models are proposed in Figure 7. During sperm shaping in spermatids, SEPT14 interacts with ACTN4 and may modulate actin function, which facilitates sperm head shaping. If sperm with mutated SEPT14 disrupt ACTN4 function and actin polymerization, sperm head formation defects occur.

### 4.4. Genetic Changes of SEPT14 in Parkinson’s Disease and Cancer

Mutations in human SEPTs have been linked to the molecular pathology of several cancers, such as leukemia, ovarian, and breast cancers [24,63]. Three research groups had previously found that genetic alterations of *SEPT14* were involved in different diseases. (1) Two SEPT14 SNPs, rs11981883, and rs10241628, were found to be associated with a reduced risk of Parkinson’s disease (PD through sequencing of 720 PD patients and 740 controls [35]. Hence, SEPT14 has been assigned a protective role, while *SEPT14* SNPs have been suggested to play a role in Parkinson’s disease pathogenesis. (2) EGFR-SEPT14 fusion has been identified in human glioblastoma through RNA-seq [64]. The EGFP-SEPT14 fusion deregulates downstream STAT3 signaling and affects the sensitivity of inhibitors in this signaling pathway. (3) A rare EGFR-SEPT14 fusion has been identified in colorectal cancer and highlights a new target for therapeutic intervention [65]. In both this and a previous study, we identified two *SEPT14* mutations (SEPT14^A123T^ and SEPT14^I333T^) in teratozoospermia cases. Furthermore, the mutation disrupts ACTN4 function, actin polymerization, and subsequently results in sperm head formation defects. However, whether these two mutations induce or promote carcinogenesis-related processes still needs to be studied.

## 5. Conclusions

In this study, we provided evidence that the molecular pathological mechanism of SEPT14-mutation-induced sperm head defects involves disruption of ACTN4-actin function during mammalian spermiogenesis.

## Figures and Tables

**Table 1:** SEPT14-interacting proteins identified by co-IP and nano-liquid chromatography-mass spectrometry/mass spectrometry.

Symbol	Gene	Functions
**SEPT-related proteins**		
*SEPT2*	Septin 2	Cell division, cilium assembly
*SEPT7*	Septin 7	Cytokinesis, cilium morphogenesis
*SEPT9*	Septin 9	Cell division
*SEPT11*	Septin 11	Cytokinesis, vesicle trafficking
**Microtubule-related proteins**		
*TUBB2A*	Tubulin beta 2A	Mitosis, intracellular transport
*TUBB2B*	Tubulin beta 2B	Mitosis, intracellular transport
*TUBB4B*	Tubulin, beta 4B	Mitosis, intracellular transport
*STMN1*	Stathmin	Microtubule depolymerization, axonogenesis
*KIF5B*	Kinesin family member 5B	Cytoskeleton-dependent intracellular transport
*DYNC1LI1*	Dynein, cytoplasmic 1, light intermediate chain 1	Cell division, microtubule-based movement
**Actin-related proteins**		
*ACTA1*	Actin, alpha 1	Cell motility, structure, and integrity
*ACTN2*	Actinin, alpha 2	Actin filament binding, muscle filament sliding, cell adhesion
*ACTN3*	Actinin, alpha 3	Crosslinking actin containing thin filaments, focal adhesion assembly
*ACTN4*	Actinin, alpha 4	Binding actin to the membrane, vesicle transport along actin filament
*CTTN*	Cortactin	Regulation of adherens-type junctions, organization of the actin structure
*CFL2*	Cofilin 2	Actin filament depolymerization, regulation of dendritic spine morphogenesis
*ITGB1*	Integrin beta-1	Cell adhesion, embryogenesis
*MYO1C*	Myosin IC	Membrane binding, lipid raft trafficking
*MYL6B*	Myosin light chain 6B	Muscle myosin
*MYH14*	Myosin, heavy chain 14	Cytokinesis, cell motility, cell polarity, axon guidance
*TPM3*	Tropomyosin 3	Provide stability to actin filaments and regulate access of other actin-binding proteins
*LAMA2*	Laminin subunit alpha 2	Major component of the basement membrane
*ZYX*	Zyxin	Modulate the cytoskeletal organization of actin bundles
**Sperm-related proteins**		
*ODF2*	Outer dense fiber of sperm tails 2	Spermatid development, maintaining the elastic structure and recoil of the sperm tail
*FSIP2*	Fibrous sheath interacting protein 2	AKAP4-interacting protein
